# Cross-sectional comparison of sociodemographic and tobacco use characteristics of U.S. adults who regularly use leading electronic nicotine delivery system (ENDS) products

**DOI:** 10.18332/tid/209827

**Published:** 2025-12-11

**Authors:** Amy L. Nyman, Katherine C. Henderson, Jeffrey Holland, David Ashley, Claire A. Spears, Jidong Huang, Scott R. Weaver

**Affiliations:** 1 Department of Health Policy and Behavioral Sciences, School of Public Health, Georgia State University, Atlanta, United States; 2 Department of Population Health Sciences, School of Public Health, Georgia State University, Atlanta, United States; 3 TRS Consulting56 LLC, Lilburn, United States

**Keywords:** e-cigarettes, Juul, Vuse Alto, cigarette smoking, FDA authorization

## Abstract

**INTRODUCTION:**

Before granting marketing authorization for electronic nicotine delivery systems (ENDS), the US Food and Drug Administration (FDA) must consider population risks and benefits associated with ENDS use. This study describes characteristics and usage patterns of individuals who use Juul or Vuse Alto to assess differences in product use.

**METHODS:**

A national, cross-sectional, online survey with US adults who use ENDS was conducted in 2022–2023 as the baseline component of a longitudinal study. Data from 503 people who regularly used either Juul (n=224) or Alto (n=279) were analyzed, including sociodemographic characteristics, cigarette smoking and quitting behaviors, ENDS use patterns, reasons for use, and risk perceptions. Chi-squared tests, ANOVA, and binary, ordinal, and multinomial logistic regression compared people who use each product.

**RESULTS:**

Those using Juul were less likely to have formerly smoked cigarettes than those using Alto (OR=0.50; 95% CI: 0.25–0.99), and those using Alto and currently smoking reported greater readiness to quit smoking cigarettes compared to those using Juul. People who used Juul and smoked cigarettes more often used Juul in places where they could not smoke compared with people using Alto. Those using Juul regularly were less likely to be over the age of 29 years (OR=0.47; 95% CI: 0.31–0.70) and more likely to come from racial/ethnic minoritized groups (34.1% vs 21.8%). People using Alto regularly consumed more e-liquid (6.6 mL vs 3.7 mL ) and those using Juul regularly used pods with higher nicotine content (OR=1.98; 95% CI: 1.25–3.14) than did those using Alto.

**CONCLUSIONS:**

We noted differences between people using Juul and Alto in sociodemographic characteristics and usage patterns of both cigarettes and ENDS. These data provide information about the potential impact of authorizing marketing of a new product on tobacco use behaviors.

## INTRODUCTION

In 2021, 4.5% of all US adults reported using electronic nicotine delivery systems (ENDS) every day or some days, with adult use highest among those aged 18–29 years (11.0%)^1^. Approximately 22% of current or former cigarette smoking adults used ENDS monthly or more often in 2022^2^. Recent years have seen major shifts in ENDS regulatory and market landscapes^3-5^; since the September 2020 deadline for manufacturers of then currently marketed ENDS products to submit premarket tobacco applications (PMTA), the US Food and Drug Administration (FDA) has reviewed and made determinations on more than 26 million applications^3^. As of July 2025, only thirty-nine ENDS, spanning four manufacturers (Logic, NJOY, Vuse, and Juul), all closed-system ENDS in either tobacco or menthol flavor, have been granted marketing authorization as they were deemed by the FDA Center for Tobacco Products (CTP) to meet the Appropriate for the Protection of Public Health (APPH) standard^6^.

Among the ENDS currently authorized are four Vuse products, which collectively have a leading 35.6% market share through tracked channels^7^. The Vuse Alto (closed pod) model, which received market authorization in 2024 in tobacco flavors, is considered the best seller^7^. Juul, a closed pod ENDS with a second-ranked US market share of 19.7%^7^, was issued a marketing denial order (MDO) in June 2022^8^, but remained on the market^9^ on appeal of the MDO until an authorization decision was issued in July 2025^6,10^. Both Juul and Alto have been among the top five selling ENDS products for the past several years^11^.

ENDS manufacturers with new products entering the market or products changed after 15 February 2007 must submit a PMTA for the FDA to review and either authorize or deny marketing^12^. In order for the FDA to provide a comprehensive review, they must consider the risks and benefits to the population as a whole, including whether a product is being used by those who currently or formerly smoked instead of those who have never smoked, and whether those currently smoking are using the product to quit smoking^4^. Use of ENDS brands and models has been shown to differ by gender, sexual orientation, race, and ethnicity, some of which may be driven by product characteristics such as variety of flavor options and nicotine levels^13,14^ and targeted promotion among specific racial, age, and sexual orientation groups^15-18^. Some of these studies found that Black and Hispanic youth and young adults were more likely to report Juul or Puff Bar as their usual brand, compared with youth and young adults who are White or of other races^13^. Black adolescents reported more exposure to and greater engagement with ENDS marketing, generally^16,17^, a finding that is not surprising given that Juul marketing has targeted Black consumers^15^. Differential appeal by demographic groups carries potential health equity implications; therefore, it is important that FDA understand who is using these products and for what reasons, how they are being used in relation to cigarette smoking, and the nature of the product characteristics such as flavors and nicotine content associated with use of each product.

Monitoring product user characteristics and usage patterns can help determine the appropriateness and public health implications of FDA authorization of these products. This study was conducted in 2022–2023, before Juul and Alto had received market authorization, in order to inform FDA consideration of the pending PMTAs. Now that these two similar products are both authorized for use, understanding the characteristics, use patterns, and perceptions of people who regularly use these products can help inform the population health implications of additional, similarly designed ENDS being authorized for marketing. Specifically, we aim to understand the potential differences between people who use Juul and Alto and whether authorization of one product may impact public health in a different way from another, including whether they are each used by different segments of the population. This study examines adults who regularly use Juul and Alto, in the US, prior to Alto’s market authorization and following a marketing denial order for Juul (since rescinded). It characterizes people who use Juul and Alto on sociodemographics, cigarette dependence and quit attempts (among those who smoke cigarettes), use patterns, perceptions, and reasons for use in order to highlight similarities and differences between those who regularly use these products.

## METHODS

### Participants and procedures

This study focuses on the cross-sectional baseline data from a longitudinal cohort study. Participant recruitment for this US national online survey took place between September 2022 and July 2023 using paid digital advertisements on Facebook, Instagram, and Craigslist. These social media platforms have been shown to be effective at recruiting people who use tobacco products who do not markedly differ sociodemographically from those recruited via other online recruitment methods^19,20^. The ads invited potential participants to complete an online screening survey hosted by Qualtrics. The survey included multiple automated fraud prevention and detection methods to prevent fraudulent entries. In total, 23665 screening surveys were completed, and 6574 screeners qualified to proceed with further verification. Eligible participants were aged ≥18 years and current US residents (verified by IP address and self-reported postal address.) Participants also needed to meet one of the following: 1) initiated use of at least one of the following then-authorized ENDS products – Logic (Power, Pro), NJOY (Ace, Daily), or Vuse (Solo, Vibe, Ciro) within the past 6 months and have used at least 5 pods/units of that product(s); or 2) currently use at least one of these then-unauthorized products – Juul, Puff (Bar, Plus, Flow, Max), or Alto and have used at least 5 pods/units of that product(s) in their lifetime. The six-month timeframe for initiation of the authorized products was determined as sufficient to recruit product users who were relatively new to the product but also allowed time for participants to become established (instead of one-time) users. Brand and model images were displayed in the screener to most accurately measure the brand/model used^21^. Participants additionally had to provide their name and contact information and submit an image of their ENDS device to verify use. Device images were reviewed by the research team and cross-referenced with Google images and previously collected images to verify participant-provided information was unique and trustworthy^22^. Participants’ emails, phone numbers, IP addresses, names, and dates of birth were cross-referenced with our database to ensure participants did not enter the study more than once.

**Figure 1:**
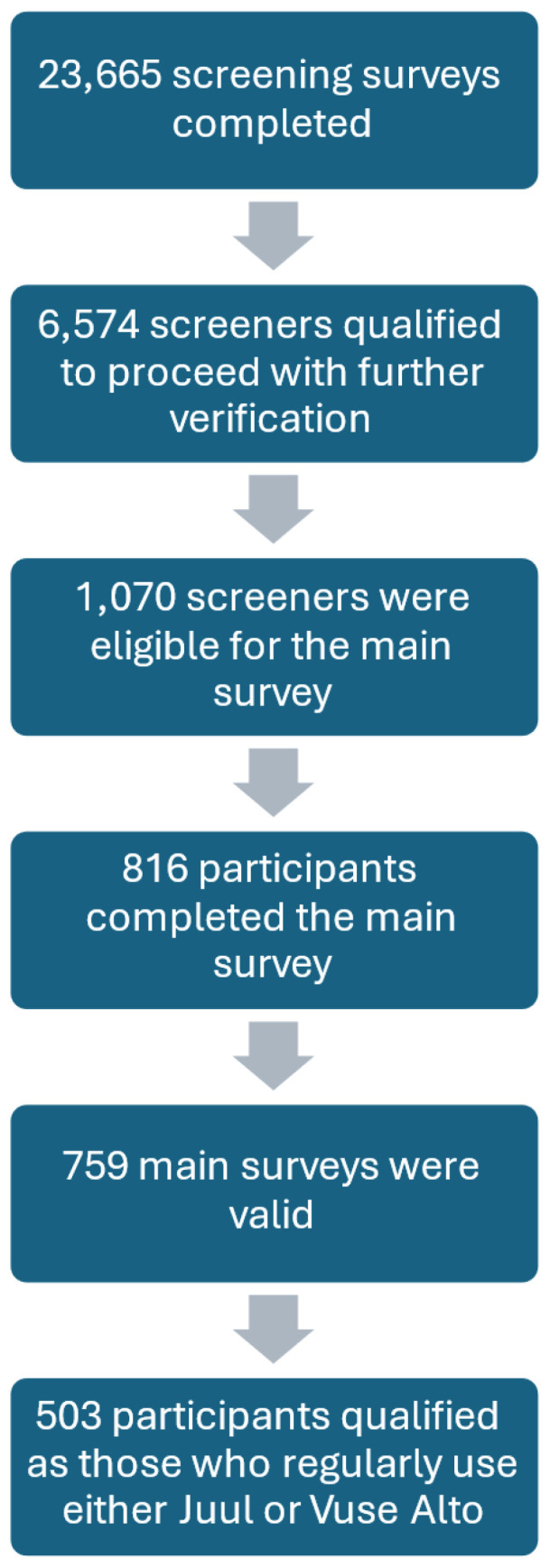
Participant flowchart from initial survey screening through qualified completed survey, US adults 2022–2023

Qualifying participants were invited 10 days later to complete the main survey, which assessed their cigarette use (smoking status, dependence, quit attempts), ENDS use patterns, perceptions, and reasons for use. Out of 1070 respondents invited to take the main survey, 816 people (76.3%) completed it. After data cleaning and quality checks were performed, 57 cases were removed for multiple screener attempts, ineligibility upon review, or having high fraud or duplicate scores as identified by Qualtrics, leaving 759 valid cases. Mutually exclusive categories were created to compare people who regularly use Juul and people who regularly use Alto who were not also regularly using the other product. In order to qualify in the ‘regular use’ group of either Juul or Alto, participants needed to meet the following criteria: 1) reported using that product at least 15 out of the past 30 days; 2) reported using one or more of that product’s pods in an average week; and 3) did not report using the other product semi-regularly (more than 4 of the past 30 days or using one or more pods in an average week). Participants who reported using both products regularly, using both products less than regularly, or using one product regularly and the other semi-regularly were excluded from analysis. Of the 759 valid cases, 503 qualified for this study as regularly using either Juul (n=224) or Alto (n=279), but not both (Figure 1). Participants received a $20 gift card for completing the main survey. This study was approved by the Georgia State University Institutional Review Board.

### Measures

Sociodemographic information was self-reported by participants and included age (18–29 vs ≥30 years), gender (cis male, cis female, non-cis), race/ethnicity (White, non-Hispanic, Black, non-Hispanic, Hispanic, Other, non-Hispanic), education level (< 4-year degree vs ≥ 4-year degree), sexual orientation (sexual minorized vs not sexual minoritized), a measure of serious psychological distress (SPD, the Kessler-6 [K6] Distress Scale)^23^, and cigarette smoking status (currently smoke every day, currently smoke some days, formerly smoked, never smoked). Responses to the K6 items were summed, with those scoring >12 considered to be experiencing SPD^23^. Among those who currently smoke cigarettes, measures included cigarette quitting efficacy (very easy; hard, but you could do it if you tried; very difficult, and you might not be able to do it; almost impossible), past year serious quit attempts (yes vs no), cigarette dependence (summative scale from four dependence items detailed in Supplementary file Table S1), and readiness to quit smoking cigarettes (0–10 scale). Measures to characterize ENDS use patterns included when participants first used their regular product (Juul or Alto) (more than 1 year ago; 1 year – 6 months ago; 6 months – 1 month ago; within 30 days), the most often used nicotine content (≤3%, 5%, other, don’t know) and most-used flavor of their regular product (menthol/mint vs tobacco/other), concurrent use ‘some days’ or ‘every day’ with other ENDS brands/models (yes vs no), use of their regular product in places where they cannot smoke cigarettes (every day, some days, rarely, never), number of days used per month, and average amount of e-liquid used per week (mL). Measures of harm perceptions and reasons for use included perceived comparative harm of ENDS compared with cigarettes (much less harmful, less harmful, about the same level of harm, more harmful, much more harmful, don’t know), use of participants’ regular ENDS product to quit smoking cigarettes (yes vs no), and the importance of each of five other statements about reasons for using their regular ENDS product (more affordable than cigarettes, can use it in places where regular cigarette smoking isn’t allowed, less harmful to me than smoking regular cigarettes, less harmful to those around me than if I smoke regular cigarettes, can help me quit smoking regular cigarettes). Complete wording and coding information for all measures can be found in Supplementary file Table S1.

### Data analysis

Percentages and means were used to describe participant characteristics, cigarette dependence and quit attempts, ENDS use patterns, harm perceptions, and reasons for use among people who regularly use Juul and Alto. To assess statistical differences, chi-squared tests (Fisher’s two-sided exact test for binary variables) were used for proportions, with binary, ordinal, or nominal logistic regressions conducted to obtain unadjusted odds ratios, and ANOVAs conducted for means, with p<0.05 considered statistically significant for all tests. The proportional odds assumption was checked for ordinal regressions, and homogeneity of variance assumption was checked for the ANOVAs. Confidence intervals (CI) for proportions were obtained using bootstrapping (bias corrected accelerated 95% intervals with 1000 simple random samples drawn with replacement). All analyses were conducted using SPSS version 29 (IBM).

## RESULTS

### Participant characteristics among those regularly using Juul or Alto

The majority of participants were cis female (58.6%) and aged ≥30 years (74.6%) (Table 1). Those regularly using Juul were less likely than those regularly using Alto to be aged >29 years (OR=0.47; 95% CI: 0.31–0.70), to identify as an individual from a sexual minoritized group (OR=0.61; 95% CI: 0.40–0.93), and to have formerly smoked cigarettes (OR=0.50; 95% CI: 0.25–0.99 vs never smoked) and more likely to have a 4-year degree (OR=2.86; 95% CI: 1.95–4.20) and to identify with a minoritized racial/ethnic group (Black NH, OR=2.02; 95% CI: 0.98–4.16; Hispanic, OR=1.78; 95% CI: 1.01–3.12; Other NH, OR=1.94; 95% CI: 1.04–3.60). No significant differences were observed for gender or serious psychological distress.

**Table 1:** Participant characteristics among US adults who regularly use^a^ Juul or Vuse Alto, 2022–2023 (N=503)

*Characteristics*	*Total* *(N=503)* *% (95% CI), n*	*Juul* *(N=224)* *% (95% CI), n*	*Vuse Alto* *(N=279)* *% (95% CI), n*	*OR^b^(95% CI)*	*p^c^*
**Age** (years)					**<0.001**
18–29	25.4 (21.9–29.1), 128	33.5 (27.5–40.1), 75	19.0 (14.1–24.2), 53	Base	
≥30	74.6 (70.5–78.4), 375	66.5 (59.5–73.0), 149	81.0 (76.0–85.7), 226	0.47 (0.31–0.70)	
**Gender**					**0.25**
Cis Male	38.2 (34.0–42.5), 192	41.1 (34.6–47.3), 92	35.8 (29.8–41.1), 100	Base	
Cis Female	58.6 (54.0–63.1), 295	54.9 (47.9–62.3), 123	61.6 (55.9–68.1), 172	0.78 (0.54–1.12)	0.18
Non-Cis	3.2 (1.9–4.8), 16	4.0 (1.8–6.8), 9	2.5 (1.1–4.2), 7	1.40 (0.50–3.91)	0.52
**Race/Ethnicity** (N=502)					**0.018**
White NH	72.9 (69.2–76.4), 366	65.9 (59.9–72.2), 147	78.5 (73.5–83.3), 219	Base	
Black NH	6.6 (4.7–8.8), 33	8.5 (5.0–12.4), 19	5.0 (2.7–8.0), 14	2.02 (0.98–4.16)	0.056
Hispanic	11.4 (8.5–14.2), 57	13.9 (9.4–18.7), 31	9.3 (5.9–12.8), 26	1.78 (1.01–3.12)	0.045
Other NH	9.2 (6.7–11.7), 46	11.7 (7.9–16.1), 26	7.2 (4.5–10.1), 20	1.94 (1.04–3.60)	0.036
**Education level**					**<0.001**
< 4-year degree	66.8 (62.6–70.9), 336	54.0 (47.2–60.4), 121	77.1 (71.6–81.9), 215	Base	
≥ 4-year degree	33.2 (29.6–37.2), 167	46.0 (39.2–52.9), 103	22.9 (18.1–28.4), 64	2.86 (1.95–4.20)	
**Sexual orientation** (N=502)					**0.023**
Sexual minoritized	25.1 (21.2–29.2), 126	20.1 (15.0–25.4), 45	29.1 (24.4–33.8), 81	0.61 (0.40–0.93)	
Not sexual minoritized	74.9 (70.9–78.7), 376	79.9 (74.5–85.0), 179	70.9 (65.0–76.5), 197	Base	
**Serious psychological distress** (score) ^d^ (N=499)					**0.38**
No (0–12)	79.0 (75.2–82.4), 394	80.9 (75.5–86.0), 178	77.4 (72.2–82.3), 216	Base	
Yes (13–24)	21.0 (17.9–24.4), 105	19.1 (14.4–24.0), 42	22.6 (17.7–27.7), 63	0.81 (0.52–1.25)	
**Cigarette smoking status**					**0.008**
Currently smoke every day	9.7 (7.4–12.1), 49	9.8 (6.3–13.6), 22	9.7 (6.5–13.3), 27	0.66 (0.28–1.55)	0.34
Currently smoke some days	27.4 (23.3–31.3), 138	33.5 (27.4–38.8), 75	22.6 (17.8–27.7), 63	0.96 (0.47–1.98)	0.92
Formerly smoked	55.3 (50.7–59.7), 278	47.3 (40.7–54.6), 106	61.6 (55.0–67.5), 172	0.50 (0.25–0.99)	0.046
Never smoked	7.6 (5.5–9.7), 38	9.4 (6.0–13.5), 21	6.1 (3.6–8.9), 17	Base	

For proportions, bias corrected accelerated bootstrapped confidence intervals are reported. Base: The reference category on the outcome variable for logistic regression models. OR: unadjusted odds ratio. NH: non-Hispanic.

a Regular use of Juul or Vuse Alto is defined as using the product ≥15 days in the past month and ≥1 pods in an average week, and not using the other product more than 4 days in the past month or ≥1 pods in an average week.

b Binary and multinomial logistic regression analyses were used to obtain unadjusted odds ratios (OR) to predict characteristics of users of each product, with Vuse Alto as the reference category.

c Bold values are from chi-squared tests of association or in the case of binary variables, Fisher’s two-sided exact tests. Non-bold values pertain to the odds ratios obtained through logistic regression.

d Kessler-6 Distress scale; scores >12 indicating a high probability of serious mental illness with significant impairment.

### Cigarette dependence and quitting among those currently smoking cigarettes and regularly using Juul or Alto

Among those who currently smoked (n=187), those regularly using Alto reported greater readiness to quit smoking than those regularly using Juul (mean=6.5; 95% CI: 5.9–7.1 vs mean=5.7; 95% CI: 5.1–6.3, on a 10-point scale). There were no significant differences in reported cigarette quitting efficacy, number of past year cigarette smoking quit attempts, or cigarette dependence (Table 2).

**Table 2:** Cigarette dependence and quit attempts among US adults currently smoking who regularly use^a^ Juul or Vuse Alto, 2022–2023 (N=187)

	*Total* *(N=187)* *% (95% CI), n*	*Juul* *(N=97)* *% (95% CI), n*	*Vuse Alto* *(N=90)* *% (95% CI), n*	*OR^b^/Mean difference* *estimate (95% CI)*	*p^c^*
**Cigarette quitting efficacy**					**0.29**
Very easy	9.6 (5.8–13.8), 18	9.3 (4.1–15.4), 9	10.0 (4.9–16.2), 9		
Hard, but you could do it if you tried	43.3 (35.6–50.7), 81	43.3 (34.3–51.6), 42	43.3 (32.6–54.5), 39	1.16 (0.68–1.99)	0.58
Very difficult, and you might not be able to do it	40.1 (33.1–47.7), 75	37.1 (27.1–46.8), 36	43.3 (32.9–52.6), 39		
Almost impossible	7.0 (3.8–10.4), 13	10.3 (5.2–16.5), 10	3.3 (0–7.5), 3		
**Past year serious cigarette quit attempt**	54.0 (47.5–61.1), 101	49.5 (39.1–59.8), 48	58.9 (48.2–69.2), 53	0.68 (0.38–1.22)	**0.24**
	***mean (95% CI), n***	***mean (95% CI), n***	***mean (95% CI), n***		
**Mean cigarette dependence^d^**	11.9 (11.3–12.4), 187	11.9 (11.1–12.7), 97	11.8 (11.0–12.6), 90	0.17 (-0.94–1.29)	**0.76**
**Mean readiness to quit smoking cigarettes^e^**	6.1 (5.7–6.5), 187	5.7 (5.1–6.3), 97	6.5 (5.9–7.1), 90	-0.84 (-1.67 – -0.012)	**0.047**

For proportions, bias corrected accelerated bootstrapped confidence intervals are reported. OR: unadjusted odds ratios.

a Regular use of Juul or Vuse Alto is defined as using the product ≥15 days in the past month and ≥1 pods in an average week, plus not using the other product more than 4 days in the past month or ≥1 pods in an average week.

b Binary or ordinal logistic regression analyses were used to obtain unadjusted odds ratios (OR) to predict characteristics of users of each product, with Vuse Alto as the reference category.

c Bold values are from the ANOVA, chi-squared tests of association or Fisher’s two-sided exact test. Non-bold values pertain to the odds ratios obtained through logistic regression.

d Summative scale with possible scores ranging from 4 to 20, with higher values signifying greater cigarette dependence.

e On a 0–10 scale; a higher number signifies greater readiness to quit smoking cigarettes.

### Use patterns of those regularly using Juul or Alto

Use patterns differed among those regularly using Juul versus Alto. Compared to those using Alto, those using Juul were less likely to have initiated use of their product more recently (OR=0.32; 95% CI: 0.21–0.49), reported using their product fewer days per month (mean=27.7; 95% CI: 27.1–28.3 vs mean=28.6; 95% CI: 28.2–29.0), and reported using nearly half the amount of e-liquid per week (3.7 mL; 95% CI: 3.3–4.1 vs 6.6 mL; 95% CI: 5.9–7.3). Those regularly using Juul also reported more use of higher nicotine content pods/cartridges (OR=1.98; 95% CI: 1.25–3.14) and more use of tobacco or other non-mint/menthol flavors (OR=1.62; 95% CI: 1.13–2.32) than those using Alto. Among people who smoked cigarettes in the past 30 days, those regularly using Juul were more likely than those regularly using Alto to report use of their product every day in places where they cannot smoke cigarettes (69.6% vs 51.9%), with Juul users being roughly half as likely to use their product in these situations less frequently (OR=0.47; 95% CI: 0.28–0.81) (Table 3).

**Table 3:** Use patterns among US adults who regularly use^a^ Juul or Vuse Alto, 2022–2023 (N=503)

	*Total* *% (95% CI), n*	*Juul* *% (95% CI), n*	*Vuse Alto* *% (95% CI), n*	*OR^b^/Mean* *difference* *estimate (95% CI)*	*p^c^*
**First time used Juul/Alto^d^** (as of screener date) (N=502)					**<0.001**
>1 year ago	72.1 (67.9–75.9), 362	83.9 (78.6–88.5), 187	62.7 (57.2–68.4), 175		
1 year – 6 months ago	9.4 (7.2–11.8), 47	6.3 (3.3–9.6), 14	11.8 (8.1–15.8), 33	0.32 (0.21–0.49)	
6 months – 1 month ago	4.2 (2.8–5.6), 21	3.6 (1.3–6.3), 8	4.7 (2.4–7.3), 13		<0.001
Within 30 days	14.3 (11.4–17.7), 72	6.3 (3.3–9.3), 14	20.8 (16.4–25.7), 58		
**Nicotine content of Juul/Alto** (N=500)					**0.010**
≤3%^e^	20.8 (17.4–24.4), 104	14.7 (9.9–19.3), 33	25.7 (20.7–30.9), 71	Base	
5%	78.4 (74.8–82.0), 392	83.9 (79.0–88.9), 188	73.9 (68.6–79.1), 204	1.98 (1.25–3.14)	0.003
Other	0.4 (0–1.0), 2	0.4 (0–1.4), 1	0.4 (0–1.2), 1	2.15 (0.13–35.5)	0.59
Don’t know	0.4 (0–1.0), 2	0.9 (0–2.3), 2	0.0 (0–0), 0	-	-
**Flavor of Juul/Alto used most often**					**0.010**
Menthol/mint	61.2 (56.9–65.6), 308	54.9 (48.2–61.3), 123	66.3 (60.5–72.1), 185	Base	
Tobacco/other	38.8 (34.8–42.9), 195	45.1 (39.0–51.6), 101	33.7 (28.6–38.9), 94	1.62 (1.13–2.32)	
**Concurrent other brand/model ENDS use^f^**					**0.93**
No	48.1 (44.2–52.2), 242	47.8 (41.5–54.6), 107	48.4 (42.6–54.2), 135	Base	
Yes	51.9 (47.5–56.5), 261	52.2 (45.4–58.6), 117	51.6 (45.8–57.4), 144	1.03 (0.72–1.46)	
Regular use of specific ENDS products^g^ (N=24)	4.8 (3.1–6.6), 24	5.8 (3.1–8.8), 13	3.9 (2.0–6.1), 11		
**Past 30-day use of Juul/Alto in places where you cannot smoke cigarettes, among those who smoked in the past 30-days** (N=221)					**0.029**
Every day	61.1 (55.2–67.4), 135	69.6 (60.4–78.2), 80	51.9 (42.9–62.0), 55		0.007
Some days	31.7 (26.2–37.6), 70	25.2 (18.3–32.8), 29	38.7 (29.3–47.4), 41	0.47 (0.28–0.81)	
Rarely	4.5 (2.3–7.2), 10	4.3 (0.9–8.4), 5	4.7 (1.1–9.3), 5		
Never	2.7 (0.9–5.0), 6	0.9 (0–2.8), 1	4.7 (1.0–9.0), 5		
	***mean (95% CI), n***	***mean (95% CI), n***	***mean (95% CI), n***		
**Days used per month among those who regularly use**	28.2 (27.9–28.6), 503	27.7 (27.1–28.3), 224	28.6 (28.2–29.0), 279	-0.93 (-1.63 – -0.23)	**0.01^i^**
**Volume in mL used per week among those who regularly use^h^**	5.3 (4.9–5.7), 503	3.7 (3.3–4.1), 224	6.6 (5.9–7.3), 279	-2.90 (-3.73 – -2.07)	**<0.001^i^**

For proportions, bias corrected accelerated bootstrapped confidence intervals are reported. Base: reference category on the outcome variable for logistic regression models.

a Regular use of Juul or Vuse Alto is defined as using the product ≥15 days in the past month and ≥1 pods in an average week, plus not using the other product more than 4 days in the past month or ≥1 pods in an average week.

b Binary, ordinal, and multinomial logistic regression analyses were used to obtain unadjusted odds ratios (OR) to predict characteristics of users of each product, with Vuse Alto as the reference category.

c Bold values are from the ANOVA, chi-squared tests of association or Fisher’s two-sided exact test. Non-bold values pertain to the odds ratios obtained through logistic regression.

d Data displayed for Juul regular users is about their Juul product, and data displayed for Vuse Alto regular users is about their Alto product.

e Juul is available in 3% and 5% nicotine, and Vuse Alto is available in 1.8%, 2.4%, and 5% nicotine.

f Concurrent ENDS use is defined as reporting ‘some day’ or ‘every day’ use of any of 13 ENDS models inquired about in our survey (Juul, Logic Power, Logic Pro, NJOY Ace, NJOY Daily, Puff Bar, Puff Plus, Puff Flow, Puff Max, Vuse Alto, Vuse Solo, Vuse Vibe, Vuse Ciro) or reporting ‘some day’ or ‘every day’ use of another type of ENDS product in addition to their regular product.

g Regular use of specific ENDS products is defined as use of any of the non-Juul, non-Alto ENDS models inquired about in our survey (Logic Power, Logic Pro, NJOY Ace, NJOY Daily, Puff Bar, Puff Plus, Puff Flow, Puff Max, Vuse Solo, Vuse Vibe, Vuse Ciro), ≥15 days in the past month and ≥1 pods in an average week.

h Volume in mL of e-liquid based on number of pods/cartridges (Vuse Alto: 1.8 mL per pod; Juul: 0.7 mL per pod).

i The assumption of homogeneity of variance was not tenable; therefore, the reported p-value is based on the Brown-Forsythe robust test of equality of means, and the confidence interval for the mean difference is adjusted.

### Harm perceptions and reasons for use among those regularly using Juul or Alto

Among people who currently or recently (past year) smoked cigarettes, those using Juul were less likely to report using their product to quit cigarette smoking (or to remain quit) than those regularly using Alto (OR=0.57; 95% CI: 0.35–0.94). Overall, the majority of people who were regularly using ENDS who also currently smoked cigarettes reported perceiving ENDS to be less harmful (52.9%; 95% CI: 45.5–60.9) or much less harmful (13.4%; 95% CI: 8.8–17.4) than cigarettes, and this did not differ statistically significantly between Alto and Juul regular use groups. Among those who currently smoked cigarettes, those regularly using Alto (vs Juul) were more likely to assign greater importance to product affordability (mean=1.36; 95% CI: 1.20–1.51 vs mean=1.08; 95% CI: 0.92–1.25) and use of their product because it is less harmful to those around them than cigarette smoking (mean=1.61; 95% CI: 1.49–1.74 vs mean=1.39; 95% CI: 1.25–1.53) (Table 4).

**Table 4:** Harm perceptions and reasons for using Juul versus Vuse Alto^a^ among US adults who currently and/or recently (past year) smoked cigarettes (2022–2023)

	*Total* *% (95% CI), n*	*Juul* *% (95% CI), n*	*Vuse Alto* *% (95% CI), n*	*OR^b^/Mean difference* *estimate (95% CI)*	*p^c^*
**Using Juul/Alto^d^ to quit cigarettes among those who currently or recently (past year) smoked** (N=324)	74.1 (69.4–78.7), 240	68.2 (60.6–75.9), 101	79.0 (72.4–85.4), 139	0.57 (0.35–0.94)	**0.031**
**Perceived comparative harm of ENDS compared with cigarettes among those who currently smoked** (N=187)					**0.33**
Much less harmful	13.4 (8.8–17.4), 25	15.5 (9.0–24.0), 15	11.1 (5.6–17.9), 10	0.71 (0.40–1.26)	
Less harmful	52.9 (45.5–60.9), 99	55.7 (45.8–65.4), 54	50.0 (39.8–60.0), 45		
About the same level of harm	23.0 (17.4–29.2), 43	19.6 (12.1–27.2), 19	26.7 (18.1–35.0), 24		0.24^e^
More harmful	2.7 (0.6–5.3), 5	4.1 (1.0–8.4), 4	1.1 (0–3.8), 1		
Much more harmful	2.1 (0.5–4.6), 4	1.0 (0–3.5), 1	3.3 (0–7.5), 3		0.77^e^
Don’t know	5.9 (2.8–9.3), 11	4.1 (1.0–8.5), 4	7.8 (2.9–13.4), 7	0.89 (0.39–2.03)	
	***mean (95% CI), n***	***mean (95% CI), n***	***mean (95% CI), n***		
**Reasons for use of your regular brand among those who currently smoked^f^**					
It is more affordable than cigarettes (N=185)	1.22 (1.10–1.33), 185	1.08 (0.92–1.25), 95	1.36 (1.20–1.51), 90	-0.27 (-0.50– -0.05)	**0.018**
I can use it in places where regular cigarette smoking is not allowed (N=186)	1.65 (1.57–1.73), 186	1.71 (1.60–1.81), 96	1.59 (1.46–1.72), 90	0.12 (-0.050 – -0.28)	**0.16^g^**
Using it is less harmful to me than smoking regular cigarettes (N=185)	1.36 (1.25–1.46), 185	1.41 (1.26–1.56), 97	1.30 (1.15–1.44), 88	0.12 (-0.09–0.32)	**0.26**
Using it is less harmful to those around me than if I smoke regular cigarettes (N=187)	1.50 (1.40–1.59), 187	1.39 (1.25–1.53), 97	1.61 (1.49–1.74), 90	-0.22 (-0.41 – -0.03)	**0.022^g^**
Using it can help me quit smoking regular cigarettes (N=185)	1.46 (1.36–1.57), 185	1.41 (1.25–1.56), 96	1.53 (1.38–1.67), 89	-0.12 (-0.33 – -0.09)	**0.26**

For proportions, bias corrected accelerated bootstrapped confidence intervals are reported. Base: reference category on the outcome variable for logistic regression models.

a Regular use of Juul or Vuse Alto is defined as using the product ≥15 days in the past month and ≥1 pods in an average week, plus not using the other product more than 4 days in the past month or ≥1 pods in an average week.

b Binary and ordinal logistic regression analyses were used to obtain unadjusted odds ratios (OR) to predict characteristics of users of each product, with Vuse Alto as the reference category.

c Bold values are from the ANOVA, chi-squared tests of association or Fisher’s two-sided exact test. Non-bold values pertain to the odds ratios obtained through logistic regression.

d Data displayed for Juul regular users is about their Juul product and data displayed for Vuse Alto regular users is about their Alto product.

e This variable was examined in two regression models: an ordinal logistic regression, excluding ‘Don’t know’ responses (OR and p-value reported in the ‘Much less harmful’ row), and as a binary logistic regression comparing ‘Don’t know’ responses with all other responses (OR and p-value reported in the ‘Don’t know’ row).

f Mean importance with ratings ranging from 0–2; higher values signify greater importance.

g The assumption of homogeneity of variance was not tenable; therefore, the reported p-value is based on the Brown-Forsythe robust test of equality of means, and the confidence interval for the mean difference is adjusted.

## DISCUSSION

Alto was authorized for marketing by US FDA in July 2024^24^, and Juul, a similar type of ENDS device, received its authorization decision in July 2025, after an initial market denial order was rescinded^10^. In deciding whether to authorize a product, FDA considers numerous factors, including the impact of product availability on population health risks and benefits, likelihood of tobacco cessation for those who currently use tobacco products and likelihood of initiation for those who do not currently use tobacco, taking into account the current product and market landscape^4^. This study examined differences in sociodemographic characteristics, smoking behavior, ENDS use patterns, and harm perceptions between adults who regularly use Juul versus Alto. These findings can inform FDA of the potential public health implications of its authorized marketing of Juul when Alto had already been authorized and is on the market, and the broader public health implications about how and by whom these products may be used differently.

Our study found people who regularly use Alto were more likely to have formerly smoked cigarettes, and among those who currently smoke cigarettes, more likely to report greater readiness to quit smoking and using Alto to quit. In contrast, among people who currently smoke cigarettes, those who regularly use Juul more often use Juul in places where they cannot smoke compared to those who regularly use Alto, a practice that may impede cigarette quitting efforts^25,26^. Some longitudinal research and randomized clinical trials have shown use of ENDS may aid cessation efforts of people who smoke who do not necessarily intend to quit^27,28^. Other studies have examined Juul specifically, with one RCT concluding use of Juul among people who smoke reduced number of cigarettes smoked^29^ and a cross-over within-subjects study found Juul use provided faster reduction of urges to smoke than some other ENDS products^30^. To our knowledge, no studies have yet compared the impact of marketing authorization of multiple ENDS products under review by the FDA. Independent cohort studies and clinical trials are needed to evaluate the impact of different ENDS products on smoking behaviors with a view toward understanding the public health implications of the overall tobacco product market and not only individual products.

Despite product design similarities, we found numerous sociodemographic differences between users of these two products, including that those who regularly use Juul tended to be younger, more educated, and more likely to come from racial/ethnic minoritized groups. The finding that more people who regularly use Juul are from racial/ethnic minoritized groups matches that of previous studies showing Juul to be more appealing to these groups^13^. Several other studies have noted Juul’s appeal among adolescents and young adults^31-33^, postulating that Juul may have been a major contributor to the concerning spike in youth e-cigarette use in 2018^34^. Our results also show those who regularly use Juul are less likely to be sexual minorities than those who regularly use Alto, suggesting the potential for differential appeal with health equity implications. To the extent that one ENDS product might be less often effectively used by those who smoke cigarettes to facilitate smoking abstinence, its disproportionate use by members of racial/ethnic minoritized groups compared to another ENDS product as an alternative could worsen health outcomes and potentially exacerbate disparities. Additional considerations could include the expanded reach to more population subgroups with additional products on the market. Further research is needed to identify marketing or other factors that might explain the differential appeal and uptake of Juul and Alto among racial/ethnic and sexual/gender minoritized groups.

Those who regularly use Alto who currently smoke cigarettes place greater importance on their product’s affordability and lower harm to those around them compared with cigarettes, which might help explain the finding that a greater proportion of people who regularly use Alto report using the product to quit smoking. Those currently/recently smoking who regularly use Juul might place greater importance on reasons that are unrelated to their cigarette smoking.

People who regularly use Alto reported consuming more mL of e-liquid but tended to use pods with lower nicotine content than those who regularly use Juul. Since Alto pods have been available in two nicotine concentrations under 3%^24^ (compared to Juul’s lowest nicotine concentration of 3%), this finding may be due in part to Alto consumers having more options available for lower nicotine content. According to the official Vuse and Juul websites at the beginning of study fielding, Alto pods contained more than double the mL of e-liquid compared with Juul, which may have contributed to the greater overall consumption of e-liquid for Alto. Regulators should further study and consider the impact of pod volume and nicotine concentration on tobacco use behaviors and outcomes.

In 2020, FDA prioritized enforcement of non-tobacco and menthol flavored pod-based ENDS and thus far has only approved tobacco flavored ENDS, with the recent exception of menthol products being authorized for NJOY’s ENDS and, most recently, Juul^10,35^. Our study revealed that a greater percentage of people who regularly use Alto (vs Juul) use menthol/mint flavors relative to tobacco or other flavors. It is possible that those in our sample who regularly use Juul had continued using flavored pods that not allowed (obtained illicitly from overseas markets or using user-modified or third-party pods) or were recalling banned flavors they had used in the past, contributing to the selection of the ‘other flavor’ category. Authorizing Juul has not included a revival of flavors other than tobacco and menthol^10^; so, the brand may have less appeal given further distance from availability of other flavors if regulators can effectively enforce bans against non-authorized pods and e-liquids.

Our study reveals trends in ENDS use that reflect the availability of these specific products. More people in our sample who regularly use Juul than regularly use Alto began using their product more than a year prior to the study. A significantly greater number of those who regularly use Alto began using Alto within 30 days before completing the survey compared with those who use Juul. These data align with Juul as the market leader versus second place Vuse in 2022 ^11^ and may indicate that those in our sample who regularly use Juul are more established in their use of Juul. However, recent 2024 market data indicate a substantial decline in market share held by Juul (19.7%) with Vuse products leading (35.6%), albeit, since 2023, also in decline as the market seems to be shifting to unauthorized disposable ENDS^7^.

### Limitations

This study has several limitations. First, we utilized a convenience sample recruited through social media, and those who may have quit smoking previously using Juul or Alto and then stopped using Juul or Alto would not have been eligible for this survey, so the findings may not be generalizable due to sampling and selection bias. Further, our results might not generalize to those who use both Juul and Vuse Alto regularly or semi-regularly as these participants were excluded from analyses due to their sample size being too small. Second, because we only asked about certain products, we do not have a complete list of all products that our sample may have been using. For example, after fielding our survey, we noted that Elf Bar (not included in our survey) was commonly used. Third, our results are dependent upon the brands and models available at the time of the survey. Fourth, while we employed numerous quality checks beyond those supplied by Qualtrics to ensure participant and data authenticity, it is possible that additional strategies may have uncovered further data quality issues^22^. Fifth, our cross-sectional, observational study is not suited for inferring causality nor was it our intention to do so. Rather, the objective of this study was to identify and compare the characteristics of the populations that use Juul or Alto, in terms of sociodemographics and tobacco/nicotine use behaviors, so that regulators and other researchers can use such information to better predict the impact of regulatory and policy actions pertaining to these products on tobacco use behaviors and health outcomes, including health disparities. While causal inference was not a necessary component of this study, some relationships identified in this study (Juul vs Alto use and smoking quitting behaviors) might be causal and could be investigated in future research designed to elucidate causal relationships and mechanisms. Finally, it should be noted that our data are self-reported and particularly for certain variables, such as nicotine concentration, may not be always accurate^36^.

## CONCLUSIONS

This study provides timely information characterizing people who regularly use two frequently used rechargeable pod-based ENDS devices: Alto and Juul. The characteristics of Juul versus Alto may appeal to different segments and may be used in different ways. Given the 2024 market authorization of Alto (in tobacco flavor), these findings shed light on the potential implications of the recent market authorization of Juul. These data may facilitate evaluation of the potential added public health impact of authorizing Juul in a marketplace where its market share has been in substantial decline. Additional consideration should be given to the impact of this decision on racial/ethnic minoritized, lower SES, and younger adult populations, and subsequent decisions for similar and different types of ENDS or other alternative consumer nicotine products (e.g. oral nicotine pouches). The findings of this study are suggestive, but not conclusive, of the possible impact and represent important considerations for FDA as they seek to reduce the harm from tobacco product use. Longitudinal research with larger national probability samples is needed to further explore and explain the findings of this study.

## Supplementary Material



## Data Availability

The data supporting this research are available from the following sources: https://doi.org/10.57709/693T-TY29
