# Supplementary material for: Cross-sectional comparison of sociodemographic and tobacco use characteristics of U.S. adults who regularly use leading electronic nicotine delivery system (ENDS) products
Source: Tob Induc Dis. 2025 Dec 11;23:10.18332/tid/209827. doi: 10.18332/tid/209827 (PMC12699319; doi:10.18332/tid/209827)
Supplement: Supplementary file 1 [file TID-23-193-s1.pdf]

Table S1. Detailed Description of Measures and Variable Construction

Construct	Question Text	Response Options	Operationalization
Study Eligibility			
Regular use of Juul or Vuse Alto	(1) On how many of the past 30 days did you use the following product(s)? [Juul]<br><br>(2) On how many of the past 30 days did you use the following product(s)? [Vuse Alto]<br><br>(3) On average, how many of the following product(s) do you use in a week? [Juul pods]<br><br>(4) On average, how many of the following product(s) do you use in a week? [Vuse Alto pods]	(1) 0 – 30<br><br>(2) 0 – 30<br><br>(3) # used<br><br>(4) # used	If (1) > 14 and (3) >= 1 and [(2) < 5 or (4) < 1], then eligible (Regular use of Juul)<br><br>Or<br><br>If (2) > 14 and (4) >= 1 and [(1) < 5 or (3) < 1], then eligible (Regular use of Vuse Alto)
Participant Characteristics			
Age	(1) What is your birthdate?<br><br>[If (1) is "Don't know", "Refused" or blank]<br>(2) About how old are you?	(1) MM/DD/YYYY<br><br>(2) Years	Variable created from (1) to age in years
Gender	(1) What terms best express how you describe your gender identity? Please select one or more.	1 = Man/Male<br>2 = Woman/Female<br>3 = Non-binary<br>4 = Transgender<br>5 = Genderqueer, Gender Nonconforming, or Genderfluid<br>6 = Agender<br>7 = None of these describe me, and I want to specify	If (1) = 1 and not 2-7, Cisgender male<br>If (1) = 2 and not 1 or 3-7, Cisgender female<br>If (1) = 8, NA<br>If (1) = else, Other

Race/Ethnicity	(1) What race or races do you consider yourself to be? Please select one or more.	(1) 1 = American Indian or Alaska Native<br>2 = Asian<br>3 = Black or African American<br>4 = Native Hawaiian or Pacific Islander<br>5 = White<br>6 = Other<br>7 = Don't know	If (2) = 1, Hispanic<br><br>If (1) = only 5 and (2) not equal to 1, White non-Hispanic<br><br>If (1) = only 3 and (2) not equal to 1, Black non-Hispanic<br><br>If (1) = else and (2) not equal to 1, Other
	(2) Do you consider yourself to be of Hispanic, Latinx, or of Spanish origin?	(2) 1 = Yes, 0 = No, 9 = Don't know	
Education	(1) What is the highest grade or level of school you have completed or the highest degree you have received?	12 = 12th grade or less, no diploma<br>13 = High school graduate<br>14 = GED or equivalent<br>15 = Some college, no degree<br>16 = Associate degree<br>17 = Bachelor's degree (Example: BA, AB, BS, BBA)<br>18 = Master's degree (Example: MA, MS, MEng, MEd, MBA)<br>19 = Professional school degree (Example: MD, DDS, DVM, JD)<br>20 = Doctoral degree (Example: PhD, EdD)	If (1) < 17, Less than 4 year degree<br><br>If (1) > 16, 4 year degree plus
Sexual Orientation	(1) Which of the following best represents how you think of yourself?	1 = Gay<br>2 = Lesbian<br>3 = Straight; that is, not gay or lesbian, etc.	If (1) = 3, Not sexual minoritized<br><br>If (1) = else, Sexual minoritized

		4 = Bisexual<br>5 = Other	
Serious Psychological Distress	During the past 30 days, about how often did you feel:<br>(1) Nervous<br>(2) Hopeless<br>(3) Restless or fidgety<br>(4) So depressed that nothing could cheer you up<br>(5) That everything was an effort<br>(6) Worthless	4 = All of the time, 3 = Most of the time, 2 = Some of the time, 1 = A little of the time, 0 = None of the time	If sum of (1), (2), (3), (4), (5), (6) < 13, No serious psychological distress<br><br>If sum of (1), (2), (3), (4), (5), (6) > 12, Serious psychological distress
Cigarette Smoking Status	(1) Have you ever smoked a cigarette, even one or two puffs?	(1) 1 = Yes, 0 = No	If (1) = 0 or (2) < 5, never smoked<br><br>If (2) = 5 and (3) = 0, formerly smoked<br><br>If (2) = 5 and (3) = 2, currently smoke every day<br><br>If (2) = 5 and (3) = 1, currently smoke some days
	(2) How many cigarettes have you smoked in your entire life? A pack usually has 20 cigarettes in it.	(2) 1 = 1 or more puffs but never a whole cigarette<br>2 = 1 to 10 cigarettes (about ½ pack total)<br>3 = 11 to 20 cigarettes (about ½ pack to 1 pack)<br>4 = 21 to 99 cigarettes (more than 1 pack but less than 5 packs)<br>5 = 100 or more cigarettes (5 packs or more)	
	(3) Do you now smoke cigarettes...	(3) 2 = Every day, 1 = Some days, 0 = Not at all	
Cigarette Dependence and Quit Attempts Among People Who Currently Smoke			

Cigarette Quitting Efficacy	If you decided you wanted to quit smoking and never start again, how easy or hard do you think it would be for you to do?	1 = Very easy<br>2 = Hard, but you could do it if you tried<br>3 = Very difficult, and you might not be able to do it<br>4 = Almost impossible	Unchanged
Past Year Serious Cigarette Quit Attempt	In the past year, have you made a serious attempt to quit smoking? That is, have you stopped smoking for at least one day or longer because you were trying to quit?	1 = Yes, 0 = No	Unchanged
Cigarette Dependence	Please rate how often each of the following statements applies to you.<br>(1) I find myself reaching for cigarettes without thinking about it.<br>(2) I drop everything to go out and buy cigarettes.<br>(3) When I haven't been able to smoke for a few hours, the craving gets intolerable.<br>(4) I smoke more before going into a situation where smoking is not allowed.	1 = Never, 2 = Rarely, 3 = Sometimes, 4 = Often, 5 = Always	Sum of (1), (2), (3), (4) with scores ranging from 4 to 20
Readiness to Quit Smoking Cigarettes	Select the number that indicates where you are now in your thinking about quitting smoking:	0 = No thought of quitting<br>1.<br>2 = Think I need to consider quitting someday<br>3.	Unchanged

		4.<br>5 = Think I should quit but not quite ready<br>6.<br>7.<br>8 = Starting to think about how to change my electronic vapor product use patterns<br>9.<br>10 = I am now taking action to quit	
ENDS Usage Patterns			
First Time Used Regular Product	When was the first time that you used [regular product]?	1 = More than a year ago, 2 = Between a year and 6 months ago, 3 = Between one month and 6 months ago, 4 = Within the past 30 days	Unchanged
Nicotine Content of Regular Product	(1) What percentage nicotine are the Juul pods you most often use?<br><br>(2) What percentage nicotine are the Vuse Altos you most often use?	(1) 1 = 3% nicotine<br>2 = 5% nicotine<br>3 = Other (Please specify)<br>9 = Don't know<br><br>(2) 1 = 1.8% nicotine<br>2 = 2.4% nicotine<br>3 = 5.0% nicotine<br>4 = Other (Please specify)<br>9 = Don't know	If (1) = 1 or if (2) = 1 or 2, 3% or less nicotine<br>If (1) = 2 or (2) = 3, 5% nicotine<br>If (1) = 3 or (2) = 4, Other<br>If (1) = 9 or (2) = 9, Don't Know
Flavor of Regular Product Used Most Often	(1) Which flavor of Juul [do] you use most often?	(1) 1 = Menthol<br>2 = Virginia Tobacco<br>3 = Some other flavor (Please specify)<br>4 = Other - Mint	If (1) = 1 or 4, or (2) = 1 or 5, Menthol/mint<br>If (1) = 2 or 3, or (2) = 2, 3, or 4, Tobacco/other

	(2) Which flavor of Vuse Alto [do] you use most often?	(2) 1 = Menthol<br>2 = Golden Tobacco flavor<br>3 = Rich Tobacco flavor<br>4 = Some other flavor (Please specify)<br>5 = Other – Mint	
Concurrent ENDS Use	(1) Do you now use the following product(s) every day, some days, or not at all?<br>Juul<br>Logic Power<br>Logic Pro<br>NJOY Ace<br>NJOY Daily<br>Puff Bar<br>Puff Plus<br>Puff Flow<br>Puff Max<br>Vuse Alto<br>Vuse Solo<br>Vuse Vibe<br>Vuse Ciro	(1) 2 = Every day, 1 = Some days, 0 = Not at all	(1) If regular use of Juul (as defined above) and<br>[(1) = Logic Power > 0 or Logic Pro > 0 or NJOY Ace > 0 or NJOY Daily > 0 or Puff Bar > 0 or Puff Plus > 0 or Puff Flow > 0 or Puff Max > 0 or Vuse Alto > 0 or Vuse Solo > 0 or Vuse Vibe > 0 or Vuse Ciro > 0]<br>Or [(2) = 3 or (3) = 3 or (4) = 3 or (5) = 3 or (7) = 3], Concurrent use<br><br>Or<br><br>If regular use of Vuse Alto (as defined above) and<br>[(1) = Juul > 0 or Logic Power > 0 or Logic Pro > 0 or NJOY Ace > 0 or NJOY Daily > 0 or Puff Bar > 0 or Puff Plus > 0 or Puff Flow > 0 or Puff Max > 0 or Vuse Solo > 0 or Vuse Vibe > 0 or Vuse Ciro > 0]
	(2) When have you used this type of electronic vapor product:<br>Cig-a-likes are electronic vapor products that mimic the size and shape of a tobacco cigarette and the nicotine is sold in pre-filled cartridges,	(2) 0 = Never used<br>1 = Have used before, but not in the past 6 months<br>2 = Used in the past 6 months, but do not currently use<br>3 = Currently use some days or every day	

capsules, or pods. Often are disposable, such as Blu Disposable, Vuse Solo, Vuse Ciro, Vuse Vibe, NJOY Daily, Logic Pro, or Logic Power.		Or [(2) = 3 or (3) = 3 or (4) = 3 or (5) = 3 or (7) = 3], Concurrent use
(3) When have you used this type of electronic vapor product:<br>Vape Pens/eGo often have the appearance of an ink pen. These are rechargeable and have a refillable tank that the user fills with a nicotine solution. Examples include Apollo Endeavor and VaporFi Rocket 3.	(3) 0 = Never used<br>1 = Have used before, but not in the past 6 months<br>2 = Used in the past 6 months, but do not currently use<br>3 = Currently use some days or every day	
(4) When have you used this type of electronic vapor product:<br>Rebuildable/mechanical mods or box mods are the largest electronic vapor product devices. They have a bigger battery, refillable tank, and are more customizable. Examples include Geekvape Legend and Vaporesso Target 100.	(4) 0 = Never used<br>1 = Have used before, but not in the past 6 months<br>2 = Used in the past 6 months, but do not currently use<br>3 = Currently use some days or every day	
(5) When have you used this type of electronic vapor product:	(5) 0 = Never used<br>1 = Have used before, but not in the past 6 months	

Rechargeable and refillable pod systems are small electronic vapor products that can look like a USB drive. Pods are refillable. Examples include Suorin Drop, Smok Novo, and Phix.	2 = Used in the past 6 months, but do not currently use<br>3 = Currently use some days or every day
(6) When have you used this type of electronic vapor product:<br>Rechargeable but not refillable pod systems: Often smaller and can look like a USB drive. Pods are purchased pre-filled and are not refillable. Examples include Juul, NJOY Ace and Vuse Alto.	(6) 0 = Never used<br>1 = Have used before, but not in the past 6 months<br>2 = Used in the past 6 months, but do not currently use<br>3 = Currently use some days or every day
(7) When have you used this type of electronic vapor product:<br>Disposable pod systems are pod-based devices designed for single use. They come fully charged and pre-filled with nicotine solution. When the e-juice is gone, they are thrown away. Examples include Posh, Puff Bar, Puff Plus, Puff Flow, Puff Max, Wave Bar, Mojo, and STIG.	(7) 0 = Never used<br>1 = Have used before, but not in the past 6 months<br>2 = Used in the past 6 months, but do not currently use<br>3 = Currently use some days or every day

Past 30-Day Use of Regular Product in Places Where Can't Smoke Cigarettes	During the past 30 days, how often did you use [regular product] at times when, or in places where you could not smoke regular cigarettes?	1 = Every day, 2 = Some days, 3 = Rarely, 4 = Never	Unchanged
Days Per Month Used Regular Product	On how many of the past 30 days did you use [regular product]?	0 – 30 days	Unchanged
Milliliters Used Per Week	(1) On average, how many of the following product(s) do you use in a week? Juul pods	(1) # used	Juul pods contain 0.7 ml per pod: (1) * 0.7
	(2) On average, how many of the following product(s) do you use in a week? Vuse Alto pods	(2) # used	Vuse Alto pods contain 1.8 ml per pod: (2) * 1.8
Harm Perceptions and Reasons for Use			
Using Regular Product to Quit Cigarettes	Are you currently using [regular product] to quit smoking regular cigarettes or to remain quit?	1 = Yes, 0 = No	Unchanged
Perceived Comparative Harm of ENDS Compared with Cigarettes	Is using electronic vapor products less harmful, about the same, or more harmful than smoking regular cigarettes?	-2 = Much less harmful<br>-1 = Less harmful<br>0 = About the same level of harm<br>1 = More harmful<br>2 = Much more harmful<br>9 = Don't know	Unchanged
Reasons for Use of Regular Brand	How important are the following reasons for your use of [regular product]?	0 = Not important, 1 = Somewhat important, 2= Very important	Unchanged

		(1) It is more affordable than cigarettes<br>(2) I can use it in places where regular cigarette smoking isn't allowed<br>(3) Using it is less harmful to me than smoking regular cigarettes<br>(4) Using it is less harmful to those around me than if I smoke regular cigarettes<br>(5) Using it can help me quit smoking regular cigarettes	

ENDS = Electronic Nicotine Delivery Systems
# = Number
NA = Not Applicable

© 2025 Nyman A.L. et al.
